# Mental health and quality of life burden in Buruli ulcer disease patients in Ghana

**DOI:** 10.1186/s40249-021-00891-8

**Published:** 2021-08-17

**Authors:** Yaw Ampem Amoako, Nancy Ackam, John-Paul Omuojine, Michael Ntiamoah Oppong, Abena Gyawu Owusu-Ansah, Harriet Boateng, Mohammed Kabiru Abass, George Amofa, Elizabeth Ofori, Portia Boakye Okyere, Michael Frimpong, Freddie Bailey, David Hurst Molyneux, Richard Odame Phillips

**Affiliations:** 1 Kumasi Centre for Collaborative Research in Tropical Medicine, Kwame Nkrumah University of Science and Technology, Kumasi, Ghana; 2 School of Medicine and Dentistry, Kwame Nkrumah University of Science and Technology, Kumasi, Ghana; 3 Komfo Anokye Teaching Hospital, Kumasi, Ghana; 4 Agogo Presbyterian Hospital, Agogo, Ghana; 5 Dunkwa Government Hospital, Dunkwa, Ghana; 6 Tepa Government Hospital, Tepa, Ghana; 7 Department of Tropical Disease Biology, Liverpool School of Tropical Medicine, Pembroke Place, Liverpool, L3 5QA UK

**Keywords:** Mental health, Depression, Anxiety, Quality of life, Buruli ulcer disease, Neglected tropical disease

## Abstract

**Background:**

Buruli ulcer disease (BUD) is a necrotic skin neglected tropical disease (NTD) that has both a mental and physical health impact on affected individuals. Although there is increasing evidence suggesting a strong association between neglected tropical diseases (NTDs) and mental illness, there is a relative lack of information on BUD’s impact on the mental health and quality of life (QoL) of affected individuals in Ghana. This study is to assess the impact of BUD on mental health and quality of life of patients with active and past BUD infection, and their caregivers.

**Methods:**

We conducted a case control study in 3 BUD endemic districts in Ghana between August and November 2019. Face-to-face structured questionnaire-based interviews were conducted on BUD patients with active and past infection, as well as caregivers of BUD patients using WHO Quality of Life scale, WHO Disability Assessment Schedule, Self-Reporting Questionnaire, Buruli Ulcer Functional Limitation Score and Hospital Anxiety and Depression Scale data tools. Descriptive statistics were used to summarize the characteristics of the study participants. Participant groups were compared using student *t* test and chi-square (*χ*^2^) or Fisher’s exact tests. Mean quality of life scores are reported with their respective 95% confidence intervals. Data was analysed using STATA statistical software.

**Results:**

Our results show that BUD patients with active and past infection, along with their caregivers, face significant levels of distress and mental health sequelae compared to controls. Depression (*P* = 0.003) was more common in participants with active (27%) and past BU infection (17%), compared to controls (0%). Anxiety was found in 42% (11/26) and 20% (6/29) of participants with active and past BUD infection compared to 14% (5/36) of controls. Quality of life was also significantly diminished in active BUD infection, compared to controls. In the physical health domain, mean QoL scores were 54 ± 11.1 and 56 ± 11.0 (95% *CI*: 49.5‒58.5 and 52.2‒59.7) respectively for participants with active infection and controls. Similarly in the psychological domain, scores were lower for active infection than controls [57.1 ± 15.2 (95% *CI*: 50.9‒63.2) vs 64.7 ± 11.6 (95% *CI*: 60.8‒68.6)]. Participants with past infection had high QoL scores in both physical [61.3 ± 13.5 (95% *CI*: 56.1‒66.5)] and psychological health domains [68.4 ± 14.6 (95% *CI*: 62.7‒74.0)].

**Conclusions:**

BUD is associated with significant mental health distress and reduced quality of life in affected persons and their caregivers in Ghana. There is a need for integration of psychosocial interventions in the management of the disease.

**Graphic abstract:**

**Figure:**
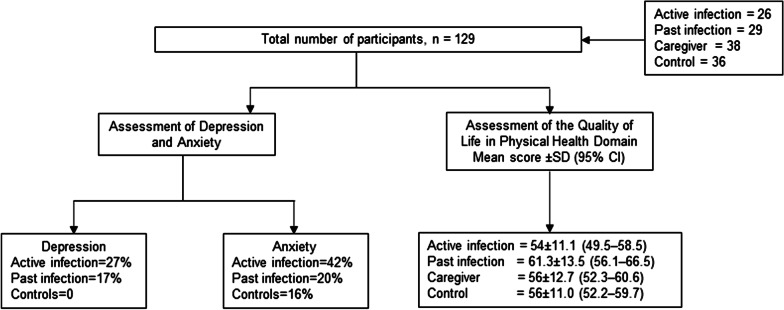

**Supplementary Information:**

The online version contains supplementary material available at 10.1186/s40249-021-00891-8.

## Background

Buruli ulcer disease (BUD) is a debilitating neglected tropical disease (NTD) of the skin caused by *Mycobacterium ulcerans* [1]*.* The disease mainly affects individuals from impoverished populations and has been reported in 33 countries, including parts of Asia, South America and the Western Pacific, although the highest burden countries are found in parts of Central and West Africa. The clinical presentation of BUD is varied and includes nodules, papules, oedemas, and ulcers. The lesions may enlarge with time to involve the bone and joints, which are critical sites affecting long-term mobility [2]. The mode of transmission of BUD remains unknown despite major advances in understanding disease mechanisms [2, 3]. BUD lesions may heal with significant scarring, leading to contractures and functional limitations, especially in the absence of appropriate early medical intervention [4].

Since 2012, there has been increasing evidence suggesting a strong association between Neglected Tropical Diseases (NTDs) and mental illness, with the wide-ranging physical and socio-economic impacts of NTDs being linked to the development of common mental disorders such as anxiety and depression [5, 6]. This evidence has recently culminated in a WHO policy manual which advocates for the need to concentrate resources on researching the psychological sequelae of NTDs and their management, and to integrate evidence-based mental health care into NTD programmes worldwide [7]. Per the WHO definition, mental health is “a state of well-being in which every individual realizes his or her own potential, can cope with the normal stresses of life, can work productively and fruitfully, and is able to make a contribution to his or her community” [8].

Functional limitations and stigmatizing scars from BUD have severe consequences on affected individuals. BUD patients have been reported to show a reduced quality of life (QoL) and high degrees of psychological co-morbidity [5]. A Ghanaian study showed that the disease had been attributed to cultural and religious beliefs such that patients are often left socially isolated [9]. The stigmatizing effect of BUD on individuals can be long lasting and patients at times are unable to interact in their communities even after being healed [10], a common finding in other chronic skin NTDs [11–13]. Functional limitation and financial burden at times require many patients to be supported by their families or loved ones (caregivers). Caregiving demands committing resources and time which may be challenging. Caregiver burden studies of NTDs have reported significant mental distress and reduced quality of life associated with performing such roles [13–17].

Although the mental health aspects of NTDs is a growing area of interest in the NTDs research community, studies to date have mainly focused on a small number of chronic skin NTDs (leprosy, cutaneous leishmaniasis, and lymphatic filariasis) [6]. Most studies related to the psychological aspects of BUD have mainly assessed illness perception and societal participation [9, 13], as well as its socio-economic burden [10, 18]. Relatively few studies have assessed the mental health burden of BUD [19, 20]. This evidence gap has necessitated the need to holistically assess the impact of BUD on mental health of patients and their caregivers. In this study, we aimed to assess the effect of BUD on mental health and QoL of patients with active and past BUD infection, and their caregivers.

## Methods

### Ethics statement

The study was approved by the Committee on Human Research, Publication and Ethics (CHRPE) of the Kwame Nkrumah University of Science and Technology (KNUST) with approval number CHRPE/AP/335/19. All participants provided written informed consent. All study procedures conformed with the principles guiding research in human subjects as set out in the Declaration of Helsinki [21].

### Study sites and participants

This was a case control study to assess the mental distress associated with BUD among patients with active infection (any manifestation), past infection (i.e., scars and/or contractures), and their caregivers. For the purposes of this study, we have defined BUD as active and past infection. A patient with active infection was defined as one who had been recently confirmed (in the preceding 3 months) to have BUD and was on treatment but whose lesion had not yet healed. BUD patients with past infection were those diagnosed within the preceding three years, who had completed a course of antibiotic therapy for BUD and whose lesions had healed with or without disability. According to the WHO report on ageing and health, ‘a caregiver provides care and support to someone else; such support may include: helping with self-care, household tasks, mobility, social participation and meaningful activities; offering information, advice and emotional support, as well as engaging in advocacy, providing support for decision making and peer support, and helping with advance care planning; offering respite services; and engaging in activities to foster intrinsic capacity’ [22]*.* In this study, a caregiver was defined as a person who provided such assistance to a BUD patient with either active or past infection. The study was conducted in three rural districts in Ghana namely; Asante Akim North, Ahafo Ano North (both districts in the Ashanti region) and Upper Denkyira East (Central region).

### Participant recruitment and sampling

BUD patients with active infection and their caregivers visiting BUD clinics for medical care were identified and recruited using a convenience sampling technique. For the recruitment of BUD patients with past infection, the hospital records of these patients were retrieved and they were contacted and informed of the study and subsequently invited to participate in their community setting. In addition, age and sex matched healthy individuals residing in BUD endemic communities were also contacted and recruited as controls using convenience sampling. Participants were provided with information leaflets informing them of the study. This was read and explained in the local language of Twi to those who were unable to read. Caregivers and healthy controls were included in the study if they had been resident in a BUD endemic community for more than two years, and were aged 18 to 60 years. Participants were excluded if they had a known physical or psychiatric illness that could confound study results. In addition, persons aged < 18 years and those unable to respond to questions were excluded. All participants provided written informed consent.

### Data tools

The data collection tools (Additional file 1) used in this study were chosen to allow comparison with the results of previous psychological [19, 20] and quality of life [4] studies of BUD, along with consideration to BUD-specific instruments and those recommended in the NTD Toolkit [23].

WHO Quality of Life-BREF (WHOQOL-BREF), is an international cross-culturally comparable QoL assessment scale used to evaluate people’s perception of their quality of life in relation to their personal goals, concerns and culture. It consists of 26 items which measure across 4 domains: physical health, psychological health, social relationships and the environment. The score for each item ranges from 1 to 5 and the total score for a domain is from 20 to 100 with higher scores indicating greater QoL [24–27].

WHO Disability Assessment Schedule (WHODAS) 2.0 and the Buruli Ulcer Functional Limitation Score (BUFLS) were employed to assess the degree of functional limitation and participation in BUD patients. The WHODAS 2.0 generally assesses functioning in 6 domains; cognition, mobility, self-care, relationships, life activities and participation in community activities [28]. Scores for each item on WHODAS 2.0 range from 0 to 4. The scores of items across each domain are computed with higher scores representing higher functional limitation.

BUFLS was designed and validated in Benin and Ghana [29] and is specifically used for assessing functional limitation in BUD. It consists of items related to the performance of 19 common daily activities. Each item is scored between 0 and 2 (0 = can perform activities easily, 1 = difficulties in performing activities and 2 = cannot perform activities at all). Functional limitation score is calculated by summing the individual item scores and dividing by the maximum possible score for an individual and finally multiplied by 100. Higher scores indicate more functional limitations with range between 0 and 100%. A score was not calculated if more than 6 items were not applicable.

Self-reporting questionnaire SRQ-20 is a 20-item scale used to screen for symptoms of mental distress. The score for each item ranges from 0 (symptom absent) to 1 (symptom present). Score items are summed to obtain the total score. Score above cut-off point indicates probable mental distress [30]. A cut-off score of 8 is widely used, however optimal cut off ranges vary across languages and settings [31–33]. In this study, we cautiously used 5 as our cut-off point to detect distress that may be present in BUD patients with pre-ulcerative forms.

Hospital Anxiety and Depression Scale (HADS) was chosen to screen for anxiety and depression among the study population. The HADS tool assesses two subscales (anxiety and depression) with scoring for each item ranging from 0 to 3 (0 = lowest anxiety or depression level; 3 = highest anxiety or depression level). A total subscale of 0–7 indicates an absence of anxiety and/or depression; scores 8–10 indicate mild symptoms of either anxiety and/or depression; scores 11–14 indicate moderate symptoms of anxiety and/or depression; scores 15–21 indicate severe symptoms of anxiety and/or depression [34].

### Tool translation

To our knowledge, the above data tools had not previously been used in the local Twi language. In keeping with WHO guidance on research tool use in different languages [35], all data tools were first translated into Twi by a professional linguistics tutor (MO) familiar with the mental health issues addressed by the various data tools. Next, the translated tools (Additional file 2) were reviewed and portions back-translated by the study coordinator (NA) and study research assistant (MNO). Minor changes were made to better reflect items in the original English language tools. All staff involved in the study then received training on questionnaire administration and conduction of interviews. The study team later discussed and tested the translated versions of the data tools on seven patients at the Agogo BUD clinic for comprehensibility, acceptability and relevance of the items. No major changes were considered necessary.

### Data collection

Data collection took place between 1st August and 30th November 2019 in the BUD clinics and communities within the selected districts in Ghana. Face-to-face interviews were conducted using the study data tools; interviews lasted between 45 and 60 min. For BUD patients with active and past infection, WHODAS, WHOQOL, SRQ-20, HADS, and BUFLS were administered, while WHOQOL, SRQ-20, and HADS were used for caregivers and controls. The HADS tool was only administered to participants who scored ≥ 5 on the SRQ-20 (screening) tool. Persons who scored < 5 were deemed unlikely to have a common mental disorder (anxiety or depression) and so were not administered the HADS tool. Interviews for patients with active infection and their caregivers were conducted in private in the BUD treatment clinic. Interviews for participants with past infection and controls were conducted in health facilities located within the communities. All interviews were conducted in private and confidentiality was maintained at all times. Scores were computed according to the scoring manual for each data tool. Results were entered in Microsoft Excel version 2013 (Microsoft Corporation, Redmond, WA, USA) before being exported to STATA (Stata Corp LP, College Station, Texas, USA) for further analysis.

### Statistical analysis

Study data was analysed using STATA version 14.0. Descriptive statistics were used to summarize the characteristics of the study participants. In addition, the degree of association was evaluated using student t test and chi-square (*χ*^2^) or Fisher’s exact tests, where appropriate, with a *P* value of ≤ 0.05 deemed to be statistically significant. Mean quality of life scores are reported with their respective 95% confidence intervals. All items on a Likert scale were assessed using mean scores. Each item response was scored into 3 different groups: ‘can do easily/at baseline level’; ‘can do with difficulty’; and ‘cannot do at all’. Scores were summarized using frequencies and percentage scores in a bar chart.

## Results

The results of this study have been reported in accordance with the STROBE checklist (Additional file 3).

### Characteristics of study participants and the occurrence of functional limitations

**Fig1:**
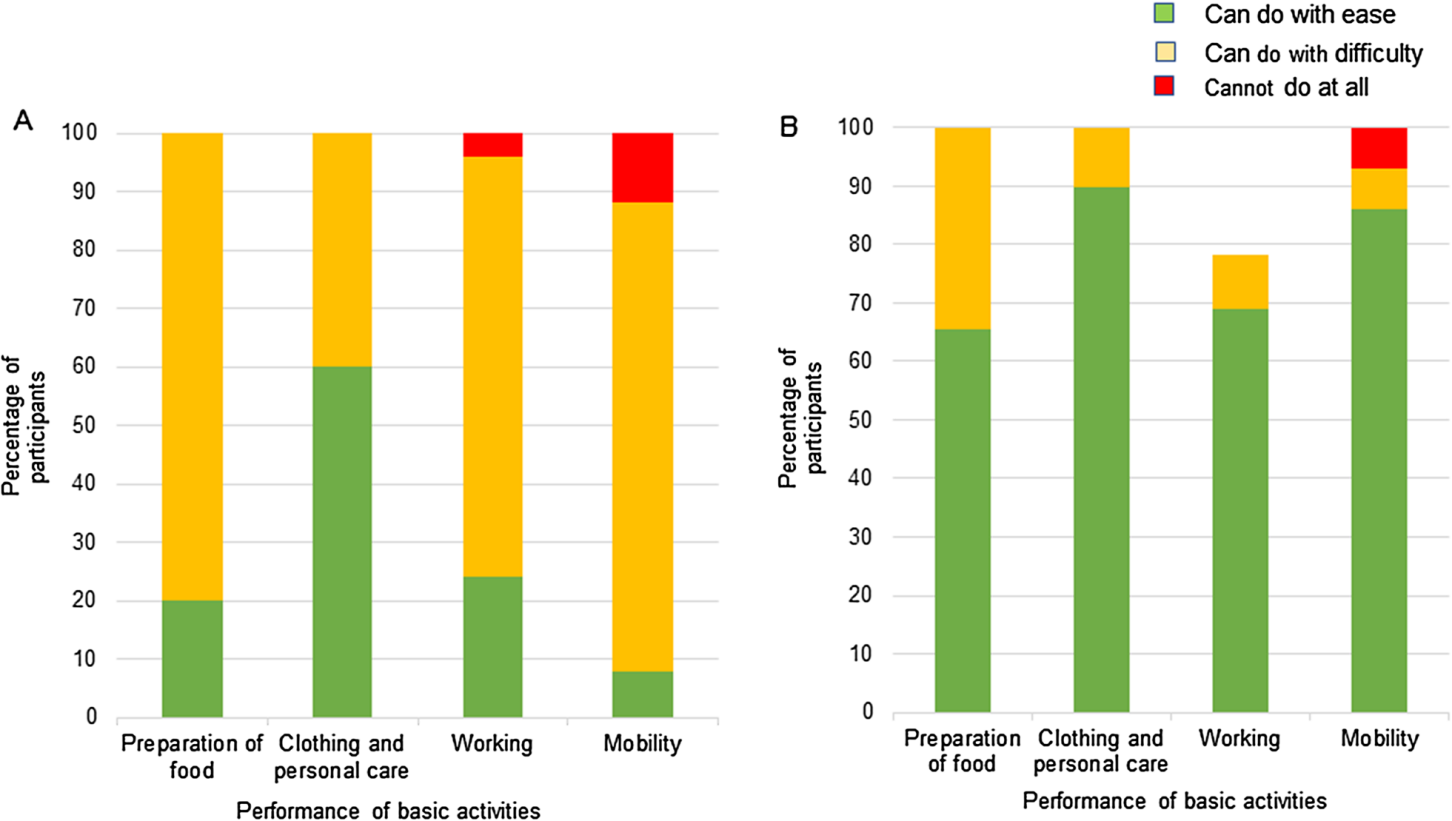
Functional limitations identified in BUD patients. **A** intensity of functional limitation faced by active BUD patients in performing basic activities. **B** Intensity of functional limitation faced by BUD patients with past infection in performing basic activities. Bar graphs show the extent of functional limitations experienced by BUD patients with active infection compared to past infection in performing basic activities. BUD: Buruli ulcer disease

In all, 129 participants consisting of 26 patients with active BU infection, 29 with past infection, 38 caregivers, and 36 controls were recruited into the study. Among the study participants, the median age was 34 years [interquartile range (IQR): 23–42], and 71 (55%) were females. 73 (65%) participants were employed, 10 (9%) were in education, and 19 (17%) were unemployed. Table 1 shows 45 (83%) BUD patients with active or past infection patients had ulcerated lesions, while 9 patients (17%) had pre-ulcerated lesions. For patients with BUD (active and past infection), lesions were most commonly located on the lower limbs in 29 (59%) individuals, with 14 (29%) having lesions on the upper limb. Six (12%) BUD patients had lesions on other parts or in multiple locations. There was significant limitation of lower limb movement in BUD patients with active infection compared to past infection (Table 2): toe movement (*P* = 0.011); knee movement (*P* = 0.028); and ankle movement (*P* = 0.011). There were no significant differences in the range of movement in the upper limbs of BUD patients with active infection when compared to past infection. Compared with past infection, more persons with active infection experienced limitation in performing basic activities (Fig. 1). The presence of pain (*P* = 0.012), ulcer (*P* = 0.0), and dry scar (*P* = 0.0) were significantly associated with functional limitation in all BUD patients (Table 3).

**Table 1:** Socio demographic and clinical characteristics of study participants

	BUD experience, *n* (%)				
Characteristics	Active infection	Past infection	Caregiver	Control	All
Number recruited	26 (20)	29 (22)	38 (30)	36 (28)	129 (100)
*Sex*					
Male	13 (50)	13 (45)	14 (37)	18 (50)	58 (45)
Female	13 (50)	16 (55)	24 (63)	18 (50)	71 (55)
Age (years)*					
≤ 20	7 (27)	6 (23)	0 (0)	4 (11)	17 (15)
21‒30	4 (15)	7 (27)	6 (24)	11 (31)	28 (25)
31‒40	4 (15)	5 (19)	8 (32)	14 (39)	31 (27)
41‒50	6 (23)	2 (8)	5 (20)	4 (11)	17 (15)
≥ 51	5 (20)	6 (23)	6 (24)	3 (8)	20 (18)
Occupation*					
Unemployed	6 (24)	8 (32)	1 (4)	4 (11)	19 (17)
Farmer	16 (64)	7 (28)	20 (80)	16 (44)	60 (54)
Artisan	0 (0)	2 (8)	0 (0)	5 (14)	7 (6)
Student	2 (8)	4 (16)	0 (0)	4 (11)	10 (9)
Other occupation	1 (4)	6 (24)	2 (8)	2 (6)	6 (5)
Lesion type*					
Ulcer	21 (81)	24 (86)	N/A	N/A	45 (83)
Oedema	2 (8)	2 (7)	N/A	N/A	4 (7)
Nodule	2 (8)	0 (0)	N/A	N/A	2 (4)
Plaque	1 (3)	2 (7)	N/A	N/A	3 (6)
Lesion location*					
Upper limb	4 (16)	10 (42)	N/A	N/A	14 (29)
Lower limb	19 (76)	10 (42)	N/A	N/A	29 (59)
Buttocks and perineum	0 (0)	1 (4)	N/A	N/A	1 (2)
Head and neck	1 (4)	0 (0)	N/A	N/A	1 (2)
Back	1 (4)	0 (0)	N/A	N/A	1 (2)
Lower limb and back	0 (0)	2 (8)	N/A	N/A	2 (4)
Upper and lower limb	0 (0)	1 (4)	N/A	N/A	1 (2)

*N/A* not applicable, *BUD* Buruli ulcer disease

*Variable contains missing data

**Table 2:** Limitation of movement in affected body part in BUD with active and past infection

Affected part	Active infection *n* = 26 (%)		Past infection *n* = 29 (%)		*P* value
	Yes	No	Yes	No	
Lower limb*					
Toe movement is reduced	10 (40)	15 (60)	3 (10)	26 (90)	0.011**
Knee movement is reduced	6 (24)	19 (76)	1 (4)	27 (97)	0.028**
Ankle movement is reduced	7 (28)	18 (72)	1 (3)	28 (97)	0.011**
Hip movement is reduced	1 (4)	24 (96)	1 (3)	28 (97)	0.92
Upper limb*					
Thumb movement is reduced	1 (4)	24 (96)	1 (3.45)	28 (97)	0.92
Wrist movement is reduced	2 (8)	23 (92)	4 (14)	25 (86)	0.50
Shoulder movement is reduced	0 (0)	25 (100)	1 (3)	28 (97)	0.35
Finger movement is reduced	2 (8)	23 (92)	2 (7)	27 (93)	0.88
Hand movement is reduced	1 (4)	24 (96)	3 (10)	26 (90)	0.38
Elbow movement is reduced	2 (8)	23 (92)	4 (14)	25 (86)	0.50

*Variable contains missing data; **P < 0.05, *BUD* Buruli ulcer disease

**Table 3:** Factors associated with functional limitation in BUD patients

Characteristics	Active infection *n* = 26 (%)	Past infection *n* = 29 (%)	*P* value
Level of education*			0.1
None	6 (26)	6 (21)	
Primary and/or middle school	17 (74)	17 (60)	
Secondary and/or above	0 (0)	5 (18)	
Dominant side*			0.08**
Right	22 (96)	22 (79)	
Left	1 (4)	6 (21)	
Pain*			0.012**
Yes	21 (91)	15 (60)	
No	2 (9)	10 (40)	
When is your pain worse*			0.17
Day	6 (26)	6 (40)	
Night	6 (26)	1 (7)	
Same all the time	11 (48)	8 (53)	
Ulcer*			0
Yes	20 (91)	2 (9)	
No	2 (9)	21 (91)	
Scar is dry*			0
Yes	1 (6)	21 (84)	
No	15 (94)	4 (16)	
Sticking / adhering scar*			0.25
Yes	4 (25)	2 (8)	
No	12 (75)	23 (92)	

*Variable contains missing data; **P < 0.05, *BUD* Buruli ulcer disease

### Assessment of the domains of quality of life

**Fig. 2:**
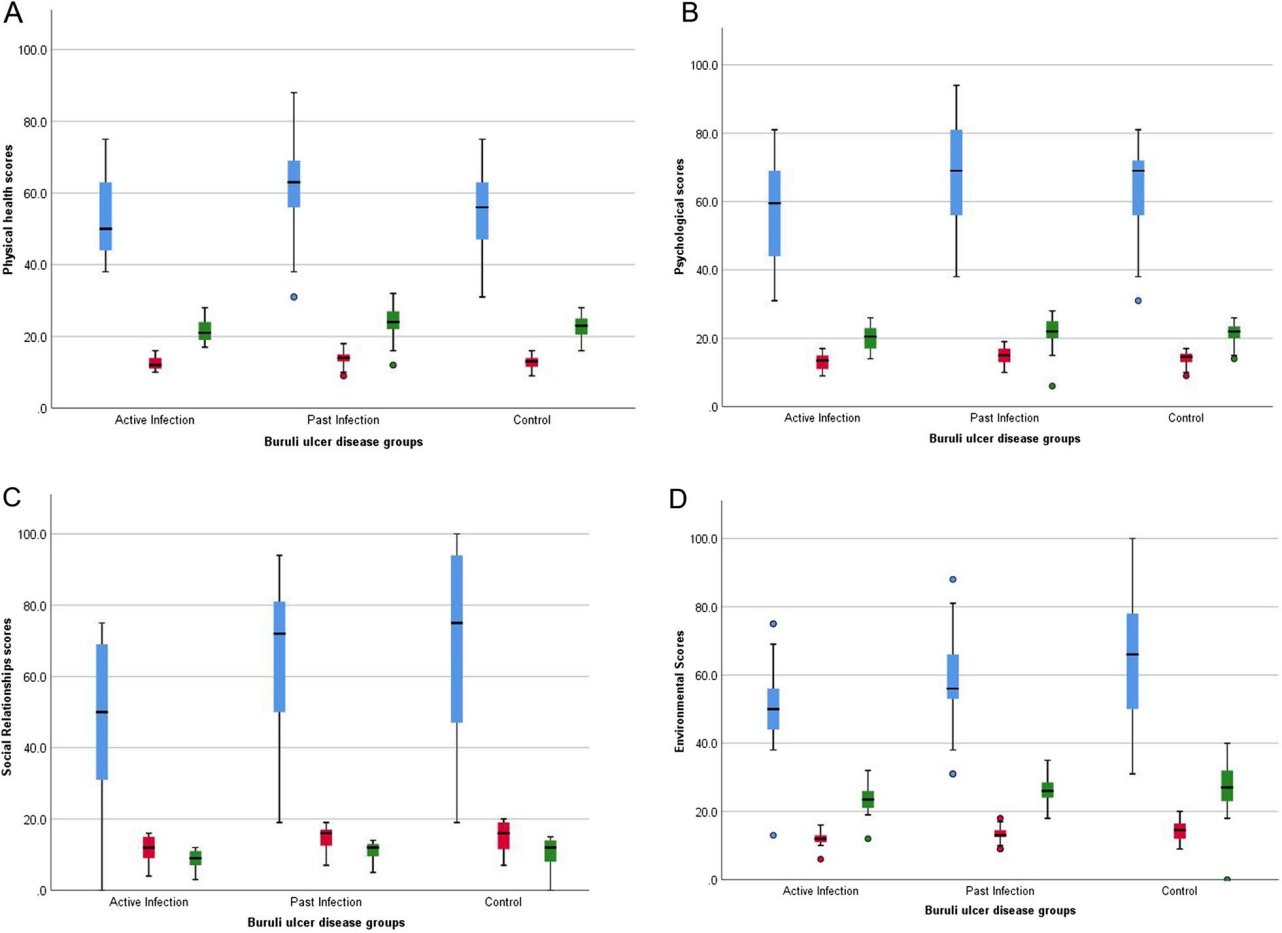
Quality of life (QoL) domain assessments of study participants. **A** Perception of QoL in physical health; **B** Perception of QoL in psychological health; **C** Perception of QoL in social relationships; **D** Perception of QoL in the environment. Box plots show the QoL assessments of study participants across the QoL domains. The box plots were constructed as median, minimum and maximum values, and interquartile ranges. The middle horizontal line represents the median distribution while the lower and upper ends of the box denote the 25th and 75th percentile of participants’ responses in each domain. The green box plots represent the raw scores while the red and blue box scores represent transformed scores of 4–20 and 0–100 respectively. BUD: Buruli ulcer disease

Figure 2 and Additional file 4 show domain scores of quality of life assessments. In the physical health domain, mean QoL scores were 54 ± 11.1 and 56 ± 11.0 (95% *CI*: 49.5‒58.5 and 52.2‒59.7) respectively for participants with active infection and controls. Similarly in the psychological domain, scores were lower for active infection than controls [57.1 ± 15.2 (95% *CI*: 50.9‒63.2) vs 64.7 ± 11.6 (95% *CI*: 60.8‒68.6)]. Participants with past infection had high QoL scores in both physical [61.3 ± 13.5 (95% *CI*: 56.1‒66.5)] and psychological health domains [68.4 ± 14.6 (95% *CI*: 62.7‒74.0)]. There was a significant difference in domains between BUD patients with active and past infection: physical health (*P* = 0.036); psychological scores (*P* = 0.007); social relationships (*P* = 0.001); and environmental (*P* = 0.001). There were also significant differences in psychological (*P* = 0.029), social relationships (*P* = 0.001) and environment domain scores (0.019) between BUD patients with active infection and healthy controls, although there was no statistical difference in the physical health domain (*P* = 0.504). Figure 2 shows higher median scores for participants with past BU infection in physical health domain compared to controls.

### Distribution and assessment of mental distress among study participants

Compared to controls, BUD patients (active and past infection) were more likely to have mental distress as assessed using the SRQ-20. Statistically significant differences were found in a total of 11/20 SRQ-20 questions, including feeling nervous (*P* = 0.001), inability to think clearly (*P* = 0.013), being unhappy (*P* = 0.001), feeling frightened (*P* = 0.013), and thinking of ending life (*P* = 0.04) (Table 4).

**Table 4:** Assessment of mental distress in study participants using SRQ-20

SRQ items	Active infection *n* = 26 (%)	Past infection *n* = 29 (%)	Control *n* = 38 (%)	*P* value
Headache	16 (62.0)	21 (72.4)	20 (55.6)	0.374
Lack of appetite	6 (23.1)	3 (10.3)	4 (11.1)	0.310
Sleeping problems	10 (38.5)	8 (27.6)	7 (19.4)	0.254
Being frightened	17 (65.4)	13 (44.8)	10 (27.8)	0.013*
Shaking hands	6 (23.1)	5 (17.2)	2 (5.6)	0.133
Feeling nervous	13 (50.0)	11 (37.9)	3 (8.3)	0.001*
Poor digestion	4 (15.4)	4 (13.8)	1 (2.8)	0.154
Not thinking clearly	9 (34.6)	4 (13.8)	2 (5.6)	0.013*
Being unhappy	16 (61.5)	12 (41.4)	6 (16.8)	0.001*
Crying more than normally	11 (42.3)	5 (17.2)	0 (0)	< 0.001*
Not enjoying activities	16 (61.5)	4 (14.3)	3 (8.3)	< 0.001*
Difficulty with decision making	9 (34.6)	6 (21.4)	5 (13.9)	0.152
Work suffering	21 (80.8)	10 (34.5)	8 (22.2)	< 0.001*
Not feeling life is useful	7 (26.9)	3 (10.3)	1 (2.8)	0.015*
Loss of interest in life	11 (42.3)	6 (20.7)	4 (11.1)	0.018*
Feeling worthless	9 (34.6)	3 (10.3)	1 (2.8)	0.002*
Thinking of ending life	6 (23.1)	1 (3.5)	2 (5.6)	0.04*
Always feeling tired	8 (30.8)	8 (27.6)	5 (13.9)	0.233
Stomach problems	14 (53.9)	11 (37.9)	8 (22.2)	0.037*
Easily tiring	11 (42.3)	9 (31.0)	8 (22.2)	0.239

*P < 0.05

The proportion of the depression and anxiety among study participants was assessed using the HADS tool. Depression (*P* = 0.003) was more common in participants with active and past BU infection, compared to controls (Table 5). Depression and anxiety were present in 4/38 (11%) and 6/38 (16%) of caregivers respectively.

**Table 5:** Distribution of anxiety and depression in study participants using HADS tool

HADS items	Active infection *n* = 26 (%)	Past infection *n* = 29 (%)	Control *n* = 36 (%)	*P* value
*Depression*				
Normal	19 (73)	24 (83)	36 (100)	0.003*
Mild	6 (23)	3 (10)	0 (0)	
Moderate/severe	1 (4)	2 (7)	0 (0)	
*Anxiety*				
Normal	15 (58)	23 (79)	31 (86)	0.136
Mild	6 (23)	3 (10)	2 (6)	
Moderate/severe	5 (19)	3 (10)	3 (8)	

*HADS* Hospital Anxiety and Depression Scale

*P < 0.05

### Relationship between functional limitation and mental disorders among BUD patients

Tables 6 and 7 show the associations between functional limitation and common mental health conditions (anxiety and depression) in patients with active and past BU infection. In participants with active BU infection, depression was linked to limitation in clothing and personal care (*P* = 0.039) (Table 6), whilst anxiety was not linked to functional limitations (Table 7). In participants with past BU infection, depression was associated with limitation in clothing and personal care (*P* = 0.048) and limitation in mobility (*P* = 0.004) (Table 6), with anxiety also being associated with limitation in mobility (*P* = 0.018) (Table 7).

**Table 6:** Comparison of association between functional limitation and occurrence of depression in BUD patients (active and past infection)

	Active infection *n* = 25 (%)				Past infection *n* = 29 (%)			
Limitation	Normal	Mild depression	Moderate/severe depression	*P* value	Normal	Mild depression	Moderate/severe depression	*P* value
*Limitation in food preparation and eating*								
Yes	13 (52)	6 (24)	1 (4)	0.3	7 (24)	2 (7)	1 (3)	0.39
No	5 (20)	0 (0)	0 (0)		17 (59)	1 (3)	1 (3)	
*Limitation in clothing and personal care*								
Yes	5 (20)	5 (20)	0 (0)	0.039*	1 (3)	1 (3)	1 (3)	0.048*
No	13 (52)	1 (4)	1 (4)		23 (79)	2 (7)	1 (3)	
*Working limitation*								
Yes	13 (52)	6 (24)	0 (0)	0.07	6 (21)	2 (7)	1 (3)	0.28
No	5 (20)	0 (0)	0 (0)		18 (62)	1 (3)	1 (3)	
*Limitation in mobility*								
Yes	16 (64)	6 (24)	1(4)	0.66	1 (3)	2 (7)	1 (3)	0.004*
No	2 (8)	0 (0)	0 (0)		23 (79)	1 (3)	1 (3)	

*P < 0.05; Hospital Anxiety and Depression Scale (HADS) scores of 0–7 = normal or no depression present, 8–10 = mild depression, 11–21 = moderate or severe depression, *BUD* Buruli ulcer disease

**Table 7:** Comparison of association between functional limitation and occurrence of anxiety in BUD patients (active and past infection)

	Active infection, *n* = 25 (%)				Past infection, *n* = 29 (%)			
Limitation	Normal	Mild anxiety	Moderate/severe anxiety	*P* value	Normal	Mild anxiety	Moderate/severe anxiety	*P* value
*Limitation in food preparation and eating*								
Yes	10 (40)	6 (24)	4 (16)	0.34	7 (24)	2 (7)	1 (3)	0.46
No	4 (16)	0 (0)	1 (4)		16 (55)	1 (3)	2 (7)	
*Limitation in clothing and personal care*								
Yes	4 (16)	3 (12)	3 (12)	0.40	1 (3.45)	1 (3.45)	1 (3.45)	0.12
No	10 (40)	3 (12)	2 (8)		22 (75.86)	1 (3.45)	1 (3.45)	
*Working limitation*								
Yes	11 (44)	3 (12)	5 (20)	0.15	6 (21)	2 (7)	1 (3)	0.36
No	3 (12)	3 (12)	0 (0)		17 (59)	1 (3)	2 (7)	
*Limitation in mobility*								
Yes	12 (48)	6 (24)	5 (20)	0.43	2 (7)	0 (0)	2 (7)	0.018*
No	2 (8)	0 (0)	0 (0)		21 (72)	3 (10)	1 (3)	

*HADS* Hospital Anxiety and Depression Scale, *BUD* Buruli ulcer disease

*P value < 0.05; HADS scores of 0–7 = normal or anxiety present, 8–10 = mild anxiety, 11–21 = moderate/ severe anxiety

## Discussion

This study holistically assessed the impact of BUD on patients (active infection as well as past infection) and their caregivers simultaneously. With these findings, we show BUD imparts substantial burden on the mental health of affected patients (active and past infection) and their caregivers when compared to controls.

In this BUD cohort, most patients had single lesions located on the lower limbs in keeping with the known epidemiology of the disease in West Africa [36]. In addition, significant levels of functional limitations in the lower limbs were observed in patients with active BU infection compared to patients with past BU infection. Factors associated with functional limitations in BUD included the presence of pain, ulcers and dry scars. Most patients with active infection presented with ulcers on the lower limbs and were more likely to have pain; this may account for the observed difference in functional limitation in the lower limbs of patients with past infection and those with active infection. Adequate management of pain is essential to minimise functional limitation in patients with active infection. Interventions aimed at preventing disability should be an integral part of BUD management as they significantly improve the overall skin condition, reduce pain, and subsequent functional limitations [37]. The Mental Wellbeing and Stigma Task Group (MWS) established by the Neglected Tropical Disease/Non-Governmental Organization/Network (NNN) encourages BUD control programmes to continually seek opportunities for integrating the mental and physical health of individuals with NTDs including BUD [6]. Such recommendations have been echoed by the WHO in its recent global health policy manual [7]. These efforts, ranging from the creation of local support groups to more formal inclusion of psychological services for affected individuals, will sustainably promote the inclusion and well-being of people living with NTDs such as BUD.

Mental distress was more prevalent in all BUD patients (active and past infection) and their caregivers, compared to controls. Depression [borderline (mild) and abnormal (moderate/severe) HADS score] was found in 27% (7/26) of active BU infection, 17% (5/26) of past BU infection, and 11% (4/38) of caregivers, compared to 0% (0/36) of controls. Anxiety [borderline (mild) and abnormal (moderate/severe) HADS score] was found in 42% (11/26) of active BU infection, 20% (6/29) of past BU infection, and 16% (6/38) of caregivers, compared to 14% (5/36) of controls. These findings for active and past BU infection are in keeping with previous psychological studies in BUD, which showed symptoms of anxiety in pre-ulcer states in 66.7% and 61.5% of ulcer states for active infection [19], and 19.5% anxiety and 31.7% depression for past infection [20].The disabling, disfiguring, and stigmatizing conditions associated with most NTDs results in mental distress in affected persons, and may result in persisting social participation restrictions even among BUD patients with past infection [10]. It is therefore not surprising that BUD patients with past infection have previously expressed a need to continue to access holistic case, including counselling services [38, 39]. Most BUD patients and caregivers showed signs of mental distress such as fear, sadness, nervousness as a result of the disease. These results are also in keeping with similar studies on mental burden of caregivers and patients with NTDs [15, 17, 19, 40].

We explored the relationship between functional limitations in BUD affected individuals and the presence of co-morbid mental disorders. From our results, a range of functional limitations were associated with anxiety and depression among BUD patients (active and past infection). Importantly, limitation in movement in past infection was associated with both anxiety and depression. One explanation for this finding may be that the scars of past infection led to an inability to move freely, impairing the participant’s ability to carry out daily activities and negatively impacting other aspects of the individual’s life, leading to reduced overall productivity and mental distress. Secondly, it was observed that patients with active infection and limitation in clothing and personal care were significantly associated with depression. It can be assumed that the necrotizing wounds on the skin of patients makes it difficult to effectively take care of themselves without external support, which could result in feelings of embarrassment and subsequent depression. Furthermore, the stigmatizing ulcers prevent patients from dressing freely without having to expose BUD affected body parts. Difficulty in mobility as well as clothing and personal care was also associated with depression in BUD patients with past infection. Early appropriate medical intervention and provision of disability prevention services have been shown to reduce functional limitation in affected body parts [1, 37]. In addition, the early diagnosis and treatment of BUD patients is likely to limit the extent of the skin lesions, thereby preventing disability, and reducing the stigma, quality of life, and mental health impact of BUD [41].

NTDs have been associated with poor QoL in affected individuals. We assessed QoL using WHOQoL based on the scores of 4 domains (physical health, psychological, social, and environmental). It was observed that active BUD participants had the lowest QoL scores out of the groups studied. QoL expressed by an individual is dependent on a host of intrinsic and extrinsic factors [42]. The disabling conditions coupled with the stigma BUD affected individuals face may have accounted for the perceived low QoL. Though caregivers are not themselves physically affected by the disease, the significant amount of time and resources they devote to caring for their sick BUD relatives, as well as the distress they may face in this role, greatly impacts their quality of life as shown in our results. Interestingly, median scores of participants with past BUD infection were higher than those of healthy controls in the physical health domain. While these differences were not statistically significant, it is still surprising given the otherwise clear trends in distress and mental health conditions in past infected patients compared to controls. The participants with past infection in this study on the whole had less functional limitations than those with active infection, which could explain why quality of life was higher than active infection. The QoL results were comparable to those in a study of former Buruli ulcer patients with early, complete treatment, and small Buruli ulcers [4]. Such results could be explained by a positive change in perspective following treatment, a psychological phenomenon known as response shift [43]. In addition, it is also possible healthy controls may have been facing some challenges unrelated to BUD, which could have influenced how they perceived their quality of life.

### Study strengths and limitations

While this study has documented significant mental distress and a high rate of anxiety and depression among BUD patients and their caregivers in three BUD endemic districts in Ghana, there were a number of limitations. Firstly, the COVID-19 pandemic resulted in disruptions to NTD services (including outreach and case detection activities) as public health resources were increasingly directed towards fighting the pandemic, which impacted recruitment into the study. Secondly, it is important to note that up to 40% of BU cases (and a significant proportion of caregivers) in West Africa are in children < 15 years old [44]. This is significant given that half of mental health conditions start by 14 years of age [45]. Nevertheless, despite children not being included in this study, findings of high mental health burden among adult patients with active and past infection persisted. Thirdly, this study was conducted in a low middle-income country and in a local language (Twi), and as such results may not be generalizable to other socio-economic and cultural settings. Additionally, information on variables such as socio-economic status/ income levels, alcohol use, tobacco use and family history of mental illness were not collected. Thus we are unable to determine the impact of such factors on the observed mental distress in participants.

Despite the limitations, this study has several strengths. This is the first study to simultaneously assess mental distress in BUD patients and their caregivers. The study included a control group of health individuals to allow for the assessment of the background levels of mental distress. Cases and controls were largely matched in terms of their demographic characteristics. Controls were selected from the communities from which BUD cases resided. Thus it is well likely that their socio-economic status would not be significantly different as their employment status were similar. However, we cannot be truly certain about this as no information on income levels were collected. Further research in other BUD endemic countries within different socio-economic settings and including younger age groups is therefore warranted.

## Conclusions

BUD results in a significant mental health and quality of life burden on both patients and caregivers alike. Evidence-based measures aimed at preventing disability and functional limitation should be encouraged in order to improve the mental health and quality of life of affected individuals. Our findings support the recommendation for integration of psychosocial interventions in BUD management for patients with active and past BU infection, as well as their caregivers.

## Supplementary Information

**Additional file 1:** Data collection tools.

**Additional file 2:** Translated data collection tools.

**Additional file 3:** STROBE checklist.

**Additional file 4:** This contains comparison tables of assessment of quality of life; association of mental disorder and functional limitation between participants with active and past BU infection and comparison of mental disorders between individuals with active BU infection and caregivers.

## Data Availability

All data generated or analysed during this study are included in this published article (and its additional information files).

## Abbreviations

BUD: Buruli ulcer disease
NTD: Neglected tropical disease
NTDs: Neglected tropical diseases
QoL: Quality of life
WHOQOL: WHO Quality of Life scale
WHODAS: WHO Disability Assessment Schedule
SRQ-20: Self-Reporting Questionnaire
BUFLS: Buruli Ulcer Functional Limitation Score
HADS: Hospital Anxiety and Depression Scale
CHRPE: Committee on Human Research, Publication and Ethics
