# Supplementary material for: Mental health and quality of life burden in Buruli ulcer disease patients in Ghana
Source: Infect Dis Poverty. 2021 Aug 17;10:109. doi: 10.1186/s40249-021-00891-8 (PMC8367773; doi:10.1186/s40249-021-00891-8)
Supplement: Supplementary file 1 — Additional file 1: Data collection tools. [file 40249_2021_891_MOESM1_ESM.pdf]

Site Number	Screening Number	Participant Study Number	Protocol Number: **T9 - 370 - 115**
**WEEK 0**			**LESION DESCRIPTION**

Examination of Lesion

Date: [__][__]/[__][__][__] /[__][__][__][__]*(DD/MMM/YYYY)*

**Lesion Type**		**Comment**
Nodule<br>Plaque<br>Oedema<br>Ulcer	[__]<br>[__]<br>[__]<br>[__]	

Measurement

Acetate tracing	[__]	Aranz equipment	[__] (please tick)
Diameter (long)	[__][__].[__] *(cm)*	Diameter (short)	[__][__].[__] *(cm)*
Surface Area:	[__][__][__].[__][__] (cm^2)	Volume	[__][__][__][__].[__][__] (cm^3)(**if Aranz**)
Healing	[__] Yes	[__] No	

If Yes,

With scab formation	[__] Yes	[__] No
Complete epithelisation	[__] Yes	[__] No
Photography	[__] Yes	[__] No

Code of digital photograph [__][__][__][__][__][__][__][__]

Shade Lesion Location

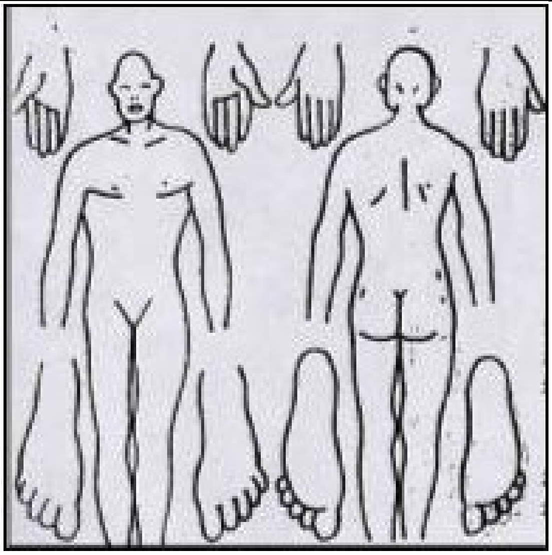

LESION LOCATION			
**Location**		**Critical Sites**	
Upper Limb (UL)<br>Lower Limb (LL)<br>Buttocks and Perineum(BP)<br>Head and Neck (HN)<br>Thorax (TH)<br>Abdomen (AB)<br>Back(BK)	[__]<br>[__]<br>[__]<br>[__]<br>[__]<br>[__]<br>[__]	Eye<br>Breast<br>Genitalia	[__]<br>[__]<br>[__]

(Fill the section below based on the Lesion location selected above e.g if Location is UPPER LIMB (UL) then fill the UPPER LIMB Section)

LIMITATIONS OF MOVEMENT OF AFFECTED BU PART			
**LOWER LIMB (LL)**			
Toe Movement is Less	Yes [__] No [__]	Ankle Movement is Less	Yes [__] No [__]
Knee Movement is Less	Yes [__] No [__]	Hip Movement is Less	Yes [__] No [__]
**UPPER LIMB (UL)**			
Thumb Movement is Less	Yes [__] No [__]	Finger Movement is Less	Yes [__] No [__]
Wrist Movement is Less	Yes [__] No [__]	Hand Movement is Less	Yes [__] No [__]
Shoulder Movement is Less	Yes [__] No [__]	Elbow Movement is Less	Yes [__] No [__]
**If OTHER, Fill the section Below**			
Trunk Movement is Less	Yes [__] No [__]		
Head/Neck Movement is Less	Yes [__] No [__]		

OEDEMA OF BU AFFECTED PART			
**LOWER LIMB (LL)**			
Toe/Foot have oedema?	Yes [__] No [__]	Knee has oedema?	Yes [__] No [__]
Ankle has oedema?	Yes [__] No [__]	Thigh has oedema?	Yes [__] No [__]
Lower leg has oedema?	Yes [__] No [__]		
**UPPER LIMB (UL)**			
Knuckles have oedema?	Yes [__] No [__]	Elbow has oedema?	Yes [__] No [__]
Wrist has oedema?	Yes [__] No [__]	Upper arm has oedema?	Yes [__] No [__]
Forearm has oedema?	Yes [__] No [__]		

Site Number	Screening Number	Participant Study Number	Protocol Number: **T9 - 370 - 115**
[ ][ ][ ]	[ ][ ][ ]	[ ][ ][ ]	
**WEEK 0**			**FUNCTIONAL LIMITATION EVALUATION**

FUNCTIONAL LIMITATION EVALUATION
Did you have a Movement Limitations Before Buruli Ulcer? Yes [__] No [__]

BU FUNCTIONAL LIMITATIONS SCORE (BUFLS)[1]		SCORE			
		2***	1**	0*	N/A****
Preparation of Food and Eating	Fetching water from pump	[__]	[__]	[__]	[__]
	Pounding Fufu /Manioc	[__]	[__]	[__]	[__]
	Pouring water from a bottle into a glass	[__]	[__]	[__]	[__]
	Cutting vegetables with a knife	[__]	[__]	[__]	[__]
Clothing and Personal care	Putting on T-Shirt	[__]	[__]	[__]	[__]
	Wash yourself	[__]	[__]	[__]	[__]
	Cleaning yourself after using toilet	[__]	[__]	[__]	[__]
Working	Using a cutlass	[__]	[__]	[__]	[__]
	Heave loads on head	[__]	[__]	[__]	[__]
	Carry harvest home	[__]	[__]	[__]	[__]
	Opening bottle with screw top	[__]	[__]	[__]	[__]
	Tie a knot	[__]	[__]	[__]	[__]
Mobility	Walking level ground	[__]	[__]	[__]	[__]
	Walking up hill	[__]	[__]	[__]	[__]
	Walking downhill	[__]	[__]	[__]	[__]
	Running	[__]	[__]	[__]	[__]
	Squatting	[__]	[__]	[__]	[__]
	Kneeling	[__]	[__]	[__]	[__]
	Standing up from floor	[__]	[__]	[__]	[__]

[1] **BURULI ULCER FUNCTIONAL LIMITATIONS SCORE:**

***N/A- Not Applicable,
***2-Cannot Do At All,
**1-Can Do with Difficulty,
*0-Can Do Easily/On Normal Level

Entered by : [__][__][__] (initial)

Other POD Assessments		
**Level of Education**		
	None [__]<br>Primary and/or Middle School Completed [__]<br>Secondary and/or above Completed [__]	
**Dominant Side**		
	Right [__]<br>Left [__]	
**Pain:**	**Yes [__] No [__]**	
**If Yes;**		
	When is your pain worse?	Day [__]<br>Night [__]<br>Same All the Time [__]
	Pain Level at the worse time (0-10)	[__][__]
	What makes your pain worse?	
	What makes your pain better?	
**Wound:**	**Yes [__] No [__]**	
**If Yes;**		
	Wound Size	[__][__].[__] cm
**Scar**		
	Hypertrophic scar present?	**Yes [__] No [__]**
	Scar is dry?	**Yes [__] No [__]**
	Scar is sticking / adhering to underlying structures?	**Yes [__] No [__]**

WHODAS 2.0

WORLD HEALTH ORGANIZATION
DISABILITY ASSESSMENT SCHEDULE 2.0

12-item version, self-administered

This questionnaire asks about difficulties due to health conditions. Health conditions include diseases or illnesses, other health problems that may be short or long lasting, injuries, mental or emotional problems, and problems with alcohol or drugs.

Think back over the past 30 days and answer these questions, thinking about how much difficulty you had doing the following activities. For each question, please circle only one response.

In the past 30 days, how much difficulty did you have in:						
S1	Standing for long periods such as 30 minutes?	None	Mild	Moderate	Severe	Extreme or cannot do
S2	Taking care of your household responsibilities?	None	Mild	Moderate	Severe	Extreme or cannot do
S3	Learning a new task, for example, learning how to get to a new place?	None	Mild	Moderate	Severe	Extreme or cannot do
S4	How much of a problem did you have joining in community activities (for example, festivities, religious or other activities) in the same way as anyone else can?	None	Mild	Moderate	Severe	Extreme or cannot do
S5	How much have you been emotionally affected by your health problems?	None	Mild	Moderate	Severe	Extreme or cannot do

Please continue to next page...

.

In the past 30 days, how much difficulty did you have in:						
S6	Concentrating on doing something for ten minutes?	None	Mild	Moderate	Severe	Extreme or cannot do
S7	Walking a long distance such as a kilometre [or equivalent]?	None	Mild	Moderate	Severe	Extreme or cannot do
S8	Washing your whole body?	None	Mild	Moderate	Severe	Extreme or cannot do
S9	Getting dressed?	None	Mild	Moderate	Severe	Extreme or cannot do
S10	Dealing with people you do not know?	None	Mild	Moderate	Severe	Extreme or cannot do
S11	Maintaining a friendship?	None	Mild	Moderate	Severe	Extreme or cannot do
S12	Your day-to-day work?	None	Mild	Moderate	Severe	Extreme or cannot do

H1	Overall, in the past 30 days, how many days were these difficulties present?	***Record number of days*** ____
H2	In the past 30 days, for how many days were you totally unable to carry out your usual activities or work because of any health condition?	***Record number of days*** ____
H3	In the past 30 days, not counting the days that you were totally unable, for how many days did you cut back or reduce your usual activities or work because of any health condition?	***Record number of days*** ____

This completes the questionnaire. Thank you.

THE WORLD HEALTH ORGANIZATION

QUALITY OF LIFE (WHOQOL) -BREF

The World Health Organization Quality of Life (WHOQOL)-BREF

© World Health Organization 2004

All rights reserved. Publications of the World Health Organization can be obtained from Marketing and Dissemination, World Health Organization, 20 Avenue Appia, 1211 Geneva 27, Switzerland (tel: +41 22 791 2476; fax: +41 22 791 4857; email: bookorders@who.int). Requests for permission to reproduce or translate WHO publications—whether for sale or for noncommercial distribution—should be addressed to Publications, at the above address (fax: +41 22 791 4806; email: permissions@who.int).

The designations employed and the presentation of the material in this publication do not imply the expression of any opinion whatsoever on the part of the World Health Organization concerning the legal status of any country, territory, city or area or of its authorities, or concerning the delimitation of its frontiers or boundaries. Dotted lines on maps represent approximate border lines for which there may not yet be full agreement.

The mention of specific companies or of certain manufacturers' products does not imply that they are endorsed or recommended by the World Health Organization in preference to others of a similar nature that are not mentioned. Errors and omissions excepted, the names of proprietary products are distinguished by initial capital letters.

The World Health Organization does not warrant that the information contained in this publication is complete and correct and shall not be liable for any damages incurred as a result of its use.

WHOQOL-BREF

The following questions ask how you feel about your quality of life, health, or other areas of your life. I will read out each question to you, along with the response options. **Please choose the answer that appears most appropriate.** If you are unsure about which response to give to a question, the first response you think of is often the best one.

Please keep in mind your standards, hopes, pleasures and concerns. We ask that you think about your life **in the last four weeks.**

		Very poor	Poor	Neither poor nor good	Good	Very good
1.	How would you rate your quality of life?	1	2	3	4	5

		Very dissatisfied	Dissatisfied	Neither satisfied nor dissatisfied	Satisfied	Very satisfied
2.	How satisfied are you with your health?	1	2	3	4	5

The following questions ask about **how much** you have experienced certain things in the last four weeks.

		Not at all	A little	A moderate amount	Very much	An extreme amount
3.	To what extent do you feel that physical pain prevents you from doing what you need to do?	5	4	3	2	1
4.	How much do you need any medical treatment to function in your daily life?	5	4	3	2	1
5.	How much do you enjoy life?	1	2	3	4	5
6.	To what extent do you feel your life to be meaningful?	1	2	3	4	5

		Not at all	A little	A moderate amount	Very much	Extremely
7.	How well are you able to concentrate?	1	2	3	4	5
8.	How safe do you feel in your daily life?	1	2	3	4	5
9.	How healthy is your physical environment?	1	2	3	4	5

The following questions ask about how completely you experience or were able to do certain things in the last four weeks.

		Not at all	A little	Moderately	Mostly	Completely
10.	Do you have enough energy for everyday life?	1	2	3	4	5
11.	Are you able to accept your bodily appearance?	1	2	3	4	5
12.	Have you enough money to meet your needs?	1	2	3	4	5
13.	How available to you is the information that you need in your day-to-day life?	1	2	3	4	5
14.	To what extent do you have the opportunity for leisure activities?	1	2	3	4	5

		Very poor	Poor	Neither poor nor good	Good	Very good
15.	How well are you able to get around?	1	2	3	4	5

		Very dissatisfied	Dissatisfied	Neither satisfied nor dissatisfied	Satisfied	Very satisfied
16.	How satisfied are you with your sleep?	1	2	3	4	5
17.	How satisfied are you with your ability to perform your daily living activities?	1	2	3	4	5
18.	How satisfied are you with your capacity for work?	1	2	3	4	5
19.	How satisfied are you with yourself?	1	2	3	4	5

20.	How satisfied are you with your personal relationships?	1	2	3	4	5
21.	How satisfied are you with your sex life?	1	2	3	4	5
22.	How satisfied are you with the support you get from your friends?	1	2	3	4	5
23.	How satisfied are you with the conditions of your living place?	1	2	3	4	5
24.	How satisfied are you with your access to health services?	1	2	3	4	5
25.	How satisfied are you with your transport?	1	2	3	4	5

The following question refers to how often you have felt or experienced certain things in the last four weeks.

		Never	Seldom	Quite often	Very often	Always
26.	How often do you have negative feelings such as blue mood, despair, anxiety, depression?	5	4	3	2	1

Do you have any comments about the assessment?

____

____

[The following table should be completed after the interview is finished]

		Equations for computing domain scores	Raw score	Transformed scores*	
				4-20	0-100
27.	**Domain 1**	(6-Q3) + (6-Q4) + Q10 + Q15 + Q16 + Q17 + Q18<br>☐ + ☐ + ☐ + ☐ + ☐ + ☐ + ☐	a. =	b:	c:
28.	**Domain 2**	Q5 + Q6 + Q7 + Q11 + Q19 + (6-Q26)<br>☐ + ☐ + ☐ + ☐ + ☐ + ☐	a. =	b:	c:
29.	**Domain 3**	Q20 + Q21 + Q22<br>☐ + ☐ + ☐	a. =	b:	c:
30.	**Domain 4**	Q8 + Q9 + Q12 + Q13 + Q14 + Q23 + Q24 + Q25<br>☐ + ☐ + ☐ + ☐ + ☐ + ☐ + ☐ + ☐	a. =	b:	c:

* See Procedures Manual, pages 13-15

Self-reporting Questionnaire (SRQ-20)

SRQ Item	YES	NO
**1.** Do you often have headaches?		
**2.** Is your appetite poor?		
**3.** Do you sleep badly?		
**4.** Are you easily frightened?		
**5.** Do your hands shake?		
**6.** Do you feel nervous, tense or worried?		
**7.** Is your digestion poor?		
**8.** Do you have trouble thinking clearly?		
**9.** Do you feel unhappy?		
**10.** Do you cry more than usual?		
**11.** Do you find it difficult to enjoy your daily activities?		
**12.** Do you find it difficult to make decisions?		
**13.** Is your daily work suffering?		
**14.** Are you unable to play a useful part in life?		
**15.** Have you lost interest in things**?**		
**16.** Do you feel that you are a worthless person?		
17. Has the thought of ending your life been on your mind		
**18.** Do you feel tired all the time?		
**19.** Are you easily tired		
**20.** Do you have uncomfortable feelings in your stomach?		
**SRQ-20 Total Score (total of yes)**		

Hospital Anxiety and Depression Scale (HADS)

Tick the box beside the reply that is closest to how you have been feeling in the past week.
Don't take too long over you replies: your immediate is best.

D	A		D	A	
		**I feel tense or 'wound up':**			**I feel as if I am slowed down:**
	3	Most of the time	3		Nearly all the time
	2	A lot of the time	2		Very often
	1	From time to time, occasionally	1		Sometimes
	0	Not at all	0		Not at all
		**I still enjoy the things I used to enjoy:**			**I get a sort of frightened feeling like 'butterflies' in the stomach:**
0		Definitely as much		0	Not at all
1		Not quite so much		1	Occasionally
2		Only a little		2	Quite Often
3		Hardly at all		3	Very Often
		**I get a sort of frightened feeling as if something awful is about to happen:**			**I have lost interest in my appearance:**
	3	Very definitely and quite badly	3		Definitely
	2	Yes, but not too badly	2		I don't take as much care as I should
	1	A little, but it doesn't worry me	1		I may not take quite as much care
	0	Not at all	0		I take just as much care as ever
		**I can laugh and see the funny side of things:**			**I feel restless as I have to be on the move:**
0		As much as I always could		3	Very much indeed
1		Not quite so much now		2	Quite a lot
2		Definitely not so much now		1	Not very much
3		Not at all		0	Not at all
		**Worrying thoughts go through my mind:**			**I look forward with enjoyment to things:**
	3	A great deal of the time	0		As much as I ever did
	2	A lot of the time	1		Rather less than I used to
	1	From time to time, but not too often	2		Definitely less than I used to
	0	Only occasionally	3		Hardly at all
		**I feel cheerful:**			**I get sudden feelings of panic:**
3		Not at all		3	Very often indeed
2		Not often		2	Quite often
1		Sometimes		1	Not very often
0		Most of the time		0	Not at all
		**I can sit at ease and feel relaxed:**			**I can enjoy a good book or radio or TV program:**
	0	Definitely	0		Often
	1	Usually	1		Sometimes
	2	Not Often	2		Not often
	3	Not at all	3		Very seldom

Please check you have answered all the questions

Scoring:
Total score: Depression (D) ___________ Anxiety (A) _____________
0-7 = Normal
8-10 = Borderline abnormal (borderline case)
11-21 = Abnormal (case)
