# Supplementary material for: Mental health and quality of life burden in Buruli ulcer disease patients in Ghana
Source: Infect Dis Poverty. 2021 Aug 17;10:109. doi: 10.1186/s40249-021-00891-8 (PMC8367773; doi:10.1186/s40249-021-00891-8)
Supplement: Supplementary file 2 — Additional file 2: Translated data collection tools. [file 40249_2021_891_MOESM2_ESM.pdf]

Nhwehwɛmu nɔma	Mfeefeemu nɔma	Deɛ wɔde no yɛɛ nhwehwɛmu no nɔma	Nhwɛhwɛmu no nɔma: **T9 - 370 - 115**
**DAPƐN 0**			**SEDEƐ PIRA NO SI TEƐ**

**Pira no ho nhwehwɛmu**
**Da:** [__][__]/[__][__][__] /[__][__][__][__]*(Da/Bosome/Afe)*

**Pira Ban / Korɔ**		**Nkasaho**
Dwoa<br>Mpomponya<br>Ahonhono<br>Akisikuro	[__]<br>[__]<br>[__]<br>[__]	

Nsusuiɛ

Acetate tracing	[__]	Aranz adwinadeɛ	[__] (Fa ahyɛnsodeɛ kyerɛ)
Mmeamu (Tentenmu)	[__][__].[__] *(cm)*	Mmeamu (Tietiamu)	[__][__].[__] *(cm)*
Animu hwɛbea :	[__][__][__].[__][__] (cm^2)	Emu duru :	[__][__][__][__].[__][__] (cm^3)(**sɛ ɛyɛ Aranz a**)
Ɛkɔ (Asa / Atɔ wo)	[__] Aane	[__] Dabi	

Sɛ Aane a,

Egya twa	[__] Aane	[__] Dabi
Ɛkɔ koraa a biribiara nni hɔ	[__]Aane	[__]Dabi
Mfoninitwa	[__]Aane	[__]Dabi

Degetaa mfonini koodu [__][__][__][__][__][__][__][__]

Fa kɔla hyɛ beaeɛ a pira no wɔ no nso.

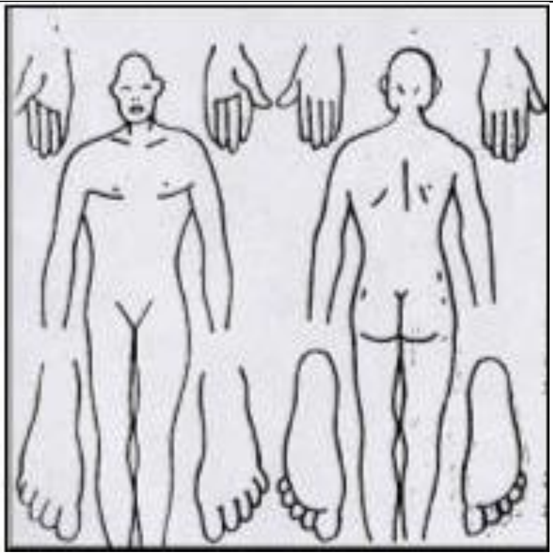

Beaeɛ a epira no wɔ			
**Beaɛ no**		**Beaeɛ a eyɛ hu**	
Nsa<br>Nnan<br>Etoɔ ne tompɔ<br>Etire ne kɔn<br>Eboɔ(kokoɔ)<br>Ayaase<br>Akyire	[__]<br>[__]<br>[__]<br>[__]<br>[__]<br>[__]<br>[__]	Ani<br>Nufoɔ<br>Awodeɛ	[__]<br>[__]<br>[__]
Gyina ɔfa a wopaeɛ wɔ atifi hɔ no so hyehye ɔfa a edi soɔ yi. Nhwɛsoɔ sɛ wofaa nsa a ,hyehyɛ nsa afa hɔ.			
**SINTƆ A EWƆ AKƆNEABADIE MU ESIANE KYEMATƆ A ABA NNIPADUA AKWAA NO BI HO ESIANE ABƆ YAREƐ NO NTI**			
**ENAN**			
Nnansoaa ahokeka wɔ fam	Aane[__] Dabi[__]	Anampɔ so ahokeka wɔ fam	Aane[__] Dabi [__]
Kotodwe ahokeka wɔ fam	Aane [__] No [__]	Dwonku no ahokekan wɔ fam	Aane [__] Dabi[__]
**Nsa**			
Kokurobetie ahokeka wɔ fam	Aane [__] Dabi __]	Nsansoa ahokeka wɔ fam	Aane [__] Dabi __]
Abakɔn ahokeka wɔ fam	Aane [__] Dabi [__]	Nsa ahokeka wɔ fam	Aane [__] Dabi __]
Abatire ahokeka wɔ fam	Aane [__] Dabi [__]	Abatwɛ ahokeka wɔ fam	Aane [__] Dabi __]
**SƐ BAABI FUFORƆ A HYEHYƐ ƆFA A EWƆ ASEƐ HƆ NO**			
Nnipadua ahokeka wɔ fam	Aane [__] Dabi [__]		
Etire / Kɔn ahokeka wɔ fam	Aane [__] Dabi [__]		
**ABƆ YAREƐ A ƐMA NNIPADUA AKWAA NO BI HONHONO**			
**Nan**			
Nansoa ne Nantabono a ɛtaataeɛ	Aane [__] Dabi [__]	Kotodwe a ɛhonhono	Aane [__] Dabi [__]
Anannpɔ a ɛhonhono	Aane [__] Dabi [__]	Asrɛ a ɛhonhono	Aane [__] Dabi [__]
Nannkoroma a ɛhonohono	Aane [__] Dabi [__]		
**Nsa**			
Nsansoa mpɔ so a ɛhonhon?	Aane [__] Dabi [__]	Abatwɛ a ɛhonhon?	Aane [__] Dabi[__]
Abakɔn a ɛhonhon??	Aane [__] Dabi [__]	Abasa a ɛhonhon?	Aane [__] Dabi[__]
Nsadua a ɛhonhon?	Aane [__] Dabi [__]		

Nhwehwɛmu nɔma			Mfeefeemu nɔma			Deɛ wɔde no yɛɛ nhwehwɛmu no nɔma		Nhwɛhwɛmu no nɔma: **T9 - 370 - 115**
**DAPƐN 0**								**APƆMUDEN HO SINTƆ MU NHWEHWƐMU**

APƆMUDEN HO SINTƆ MU NHWEHWƐMU	
Na wowɔ nnipadua mu ɔhaw bi fa wo daa daa dwumadie mu ansa na Abɔ yareɛ no rebɛyɛ wo?	Aane [__] Dabi [__]

Nnipadua mu sintɔ ho akontabuo		MMA 2***	1**	0*	E/A****
Aduanenoa Ne Adidie	Wode w'ahoɔden rebu nsuo wɔ dorobɛn mu	[__]	[__]	[__]	[__]
	Fufuo wɔ	[__]	[__]	[__]	[__]
	Worehwie nsuo afiri toa mu agu gelaase mu	[__]	[__]	[__]	[__]
	Wode sekanmoa retwitwa atosodeɛ	[__]	[__]	[__]	[__]
Ntadehyɛ ne Nipadua mu Asiesie	Ɔhyɛ "T – Hyɛɛte"	[__]	[__]	[__]	[__]
	Woredwareɛ	[__]	[__]	[__]	[__]
	Woakɔ agyananbea aba na woresiesie wo ho	[__]	[__]	[__]	[__]
Adwuma yɛ	Sɛ wode sekan reyɛ adwuma	[__]	[__]	[__]	[__]
	Worepagya nnoɔma asoa	[__]	[__]	[__]	[__]
	Woresoa nnɔbae afiri afuom abab fie	[__]	[__]	[__]	[__]
	Worebue toa a yɛkyim no ansa na abue so	[__]	[__]	[__]	[__]
	Worebɔ pɔ	[__]	[__]	[__]	[__]
Mpasatuo	Wonam asase a ɛda fam soɔ	[__]	[__]	[__]	[__]
	Woreforo bepɔ	[__]	[__]	[__]	[__]
	Woresane bepɔ	[__]	[__]	[__]	[__]
	Mmirikatuo	[__]	[__]	[__]	[__]
	Worekotoɔ	[__]	[__]	[__]	[__]
	Nkotodwebuo	[__]	[__]	[__]	[__]
	Woresɔre agyina hɔ	[__]	[__]	[__]	[__]

[1] ABƆ YAREƐ HO NKONTABUO:

***E/A- Enyɛ Adwuma,
***2-Ntumi nyɛ koraa,
**1-Ɔbɛyɛ na ɔbɛbrɛ ,
*0- Ɔbɛyɛ a ɔnte brɛ

Entered by : [__][__][__] (initial)

Nhwehwɛmu Foforo		
**Adesua mu Gyinapɛm**		
	Ɔnkɔɔ bi [__]<br>Mfitiaseɛ nteteɛɛ ne / anaa mfinimfini nteteeɛ [__]<br>Ntoasoɔ sukuu ne / anaa deɛ ɛboro saa na owieeɛ [__]	
**Deɛ n'ahoɔden gyina**		
	Nifa [__]<br>Benkum [__]	
**Yaw:**	**Aane [__] Dabi [__]**	
**Sɛ Aane a;**		
	Berɛ bɛn na yaw no yɛ kɛse?	Anɔpaa / Animteɛ [__]<br>Anadwo [__]<br>Ɛte saa berɛ biara [__]
	Ɔyaw no anoden, berɛ a ayɛ kɛseɛ no (0-10)	[__][__]
	Deɛn na ɛma woyaw no ano den?	
	Deɛn na ɛma woyaw no ano berɛ ase kakra?	
**Epira :**	**Aane [__] Dabi[__]**	
**Sɛ Aane a ;**		
	Ne kɛseɛ	[__][__].[__] cm
**Kutwa**		
	Kutwa wɔ hɔ?	**Aane [__] Dabi[__]**
	Kutwa anim wesee?	**Aane [__] Dabi[__]**
	Kutwa no aka atare honam no anim dendennen?	**Aane [__] Dabi[__]**

WHODAS 2.0

WORLD HEALTH ORGANIZATION

DƐMDIFOƆ NHWEHWƐMU HO NHYEHYƐEƐ 2.0

NSƐMMUSA -12 HO AKONTABUO, ANKORƐANKORƐ NA ƐDE MMAUAE

Saa nsɛmmisa yi fa nsɛnnennnen a apɔmudensɛm na ɛde ba ho. Nnoɔma a efa apɔmmudensɛm ho bi ne yaredɔm, nyarewa ahodoɔ Akwanhosan ho haw a ɛbetumi ayɛ bere tiaa bi anaa koankoro, pira, adwenemhaw anaa nkate mu haw ne nsanom ne nnubɔne ho nsɛnnennen ahodoɔ.

Susu bɛyɛ nnafua 30 a atwam ho na bua saa nsɛmmisa yi, na susu ɔhaw dodoɔ a wofaa mu berɛ a woredi saa dwuma yi no. Asɛmmisa biara, mesrɛ wo twa mmuaeɛ baako pɛ ho kanko.

Bɛyɛ nnafua 30 a abɛsene kɔ ɔhaw bɛn na wofaa mu wɔ:						
S1	Sɛ wogyina ntenten berɛ tenten bɛyɛ sɛ simma 30 a?	Bibiaa nni hɔ saa	Ano yɛ mmmerɛ	Ɛhɔ ne hɔ	Ano yɛ den	Ɛboro so Anaa wontumi nnyɛ
S2	Woreyɛ wo fie nnwuma a?	Bibiaa nni hɔ saa	Ano yɛ mmmerɛ	Ɛhɔ ne hɔ	Ano yɛ den	Ɛboro so Anaa wontumi nnyɛ
S3	Sɛ woresua nnwuma foforɔ, sɛ nnhwɛsoɔ, woresua sɛdeɛ wobetu akɔ atenaeɛ foforɔ?	Bibiaa nni hɔ saa	Ano yɛ mmmerɛ	Ɛhɔ ne hɔ	Ano yɛ den	Ɛboro so Anaa wontumi nnyɛ
S4	Ɔhaw ahodoɔ bɛn na wohyiaeɛ berɛ a wode woho hyɛɛ kwasafodwuma ahodoɔ mu no sɛ nnhwɛsoɔ: afahyɛ, Nyamesom ne nnwuma afoforɔ mu no sɛdeɛ nnipa foforɔ biara bɛtumi ayɛ no?	Bibiaa nni hɔ saa	Ano yɛ mmmerɛ	Ɛhɔ ne hɔ	Ano yɛ den	Ɛboro so Anaa wontumi nnyɛ
S5	W'akwanhosan a ɛnnyɛ papa ama wo ahaw akoduro hen?	Bibiaa nni hɔ saa	Ano yɛ mmmerɛ	Ɛhɔ ne hɔ	Ano yɛ den	Ɛboro so Anaa wontumi nnyɛ

Mesrɛ wo toa so wɔ kratafa a edi hɔ no…..

Bɛyɛ nnafua 30 a abɛsene kɔ,ɔhaw bɛn na wofaa mu wɔ:						
S6	Sɛ wode w'adwene resi biribi so ayɛ no bɛyɛ simma du?	Bibiaa nni hɔ saa	Ano yɛ mmmerɛ	Ɛhɔ ne hɔ	Ano yɛ den	Ɛboro so Anaa wontumi nnyɛ
S7	Worenante kwan tenten bɛyɛ sɛ borɔfo kwansin baako (anaa deɛ ɛte saa)?	Bibiaa nni hɔ saa	Ano yɛ mmmerɛ	Ɛhɔ ne hɔ	Ano yɛ den	Ɛboro so Anaa wontumi nnyɛ
S8	Sɛ worehohoro wo ho nyianaa?	Bibiaa nni hɔ saa	Ano yɛ mmmerɛ	Ɛhɔ ne hɔ	Ano yɛ den	Ɛboro so Anaa wontumi nnyɛ
S9	Sɛ woresiesie wo ho ?	Bibiaa nni hɔ saa	Ano yɛ mmmerɛ	Ɛhɔ ne hɔ	Ano yɛ den	Ɛboro so Anaa wontumi nnyɛ
S10	Wo ne nkurɔfoɔ a wonnim wɔn resan ?	Bibiaa nni hɔ saa	Ano yɛ mmmerɛ	Ɛhɔ ne hɔ	Ano yɛ den	Ɛboro so Anaa wontumi nnyɛ
S11	Sɛ worekora wo ne afoforo ayɔnkofa so?	Bibiaa nni hɔ saa	Ano yɛ mmmerɛ	Ɛhɔ ne hɔ	Ano yɛ den	Ɛboro so Anaa wontumi nnyɛ
S12	Wo daa daa adwumayɛ mu?	Bibiaa nni hɔ saa	Ano yɛ mmmerɛ	Ɛhɔ ne hɔ	Ano yɛ den	Ɛboro so Anaa wontumi nnyɛ

H1	Sɛ yɛka nnafua 30 no nyinaa bom a nna dodoɔ sɛn na na saa ɔhaw no wɔ hɔ?	Twerɛ nnafua no wɔ ha ________
H2	Nnafua 30 no ntɛm no emu nna dodoɔ sɛn na akwanhosan ho nsɛnnennen nti wantumi anni dwuma a wodi no daa no?	Twerɛ nnafua no wɔ ha ________
H3	Nnafua 30 no mu, sɛ nna dodoɔ a wantumi anni wo dwuma no da nkyɛn a, nna dodoɔ sɛn na wotee wo dwumadie a wodi mod aa no so ɛsiane apɔmudensɛm nti ?	Twerɛ nnafua no wɔ ha ________

Nsɛmmisa no awieeɛ nie. Meda wo ase.

THE WORLD HEALTH ORGANIZATION

QUALITY OF LIFE (WHOQOL) -BREF

AMANSAN APƆMUDEN KUBAATAN ASETENA – PA

WHOQOL-BREF

Nsɛmmisa ahodoɔ a edidi soɔ yi rehwehwɛ biribi afa sɛdeɛ wote nka fa asetena a ɛyɛ papa, apɔmuden anaa abrabɔ afa foforɔ ho. Mɛkan nsɛmmisa no mu biara akyerɛ wo ne wɔn mmuae ahodoɔ no, **mesrɛ wo yi mmuae a wosusu sɛ ɛfata pa ara no**. sɛ w'adwene nsi wo pi wɔ asɛmmisa mmuaeɛ ho a mmuaeɛ a edi kan ba w'adwenem no taa yɛ deɛ ɛyɛ kyen biara. Mesrɛ wo ma wo gyinapɛn ahodoɔ, anidasoɔ nnoɔma a wobu no anigyedeɛ ne nnoɔma ɛde ɔhaw berɛ wo nyinaa ntena wadwenem. Yɛsrɛ sɛ ma saa nsɛmmisa yi mmuaeɛ nnyina w'ankasa w'asetena so **bɛyɛ adapɛn nan a atwam yi ara so**.

		Ɛnyɛ koraa	Ɛnyɛ	Ɛno a ɛnsɛe na ɛno nso a ɛnyɛ papa	Ɛyɛ	Ɛyɛ pa ara
1.	Sɛ wode w'asetena to nsania ani a sɛn na ɛbɛsi atwe?	1	2	3	4	5

		M'ani nsɔ koraa	M'ani nsɔ	Ɛyɛ hɔ ne nɔ	M'ani sɔ	M'ani sɔ pa ara
2.	Sɛn na w'ani gye w'apɔmuden ho kɔpem?	1	2	3	4	5

Nsɛmmisa a edidi soɔ yi fa nnoɔma bi ho suahunu a woanya no adapɛn nan (bosome) a atwam yi mu ho.

		Ɛbi ansi da	Kakra bi	Ɛhɔ ne hɔ	Mpɛn pii	Ɛboro so
3.	Mpɛn ahen na honam yaa nka a woteɛ nti amma wantumi anni dwuma a na ɛsɛ sɛ wodie?	5	4	3	2	1
4.	Mpɛn dodoɔ sɛn nan a wohia nnuruyɛ mu ayaresa na ama wantumi ayɛ deɛ ɛyɛ sɛ woyɛ daa?	5	4	3	2	1
5.	Mpɛn dodoɔ sɛn na w'ani agye wɔ w'asetena mu?	1	2	3	4	5
6.	Sɛn na wote nka sɛ w'abrabɔ yɛ deɛ mfasoɔ wɔ soɔ?	1	2	3	4	5

		Ɛbi ansi da	Kakra bi	Ɛhɔ ne hɔ	Mpɛn pii	Ɛboro so
7.	Sɛn na wotumi de w'adwen si w'asetena mu dwumadie so yie?	1	2	3	4	5

8.	Sɛn na wosi te bammɔ nka wɔ wo daa daa asetena mu?	1	2	3	4	5
9.	Wo mpɔtɛm hɔ ahonmidie te sɛn?	1	2	3	4	5

Nsɛmmisa yi a edidi soɔ yi fa sɛdeɛ wosi dii nnoɔma binom ho dwuma yie ho nnawɔtwe nnan a atwam yi mu.

		Ɛbi ansi da	Kakra bi	Ɛyɛ	Mpɛn pii	Berɛ biara
10.	Wowɔ ahoɔden a edi mu wɔ daa daa asetena mu?	1	2	3	4	5
11.	Sɛdeɛ wo nnipadua teɛ no w'ani sɔ?	1	2	3	4	5
12.	Wowɔ sika a etumi so w'ahia deɛ?	1	2	3	4	5
13.	Nnoɔma ho nimdeɛ a wohia wɔ wo daa daa asetena mu no wo nsa ka?	1	2	3	4	5
14.	Mpɛn dodoɔ sɛn na wonya adagye de yɛ nnoɔma a wo ara wopɛ?	1	2	3	4	5

		Ɛnyɛ koraa	Ɛnyɛ	Ɛhɔ ne hɔ	Ɛnyɛ	Ɛyɛ pa ara
15.	Sɛn na wo dabiara da akɔneabadie teɛ?	1	2	3	4	5

		Very dissatisfied	Dissatisfied	Neither satisfied nor dissatisfied	Satisfied	Very satisfied
16.	Wutumi da yie pa ara?	1	2	3	4	5
17.	Tumi a wo tumi de yɛ wo daa daa asetena mu dwumadie no sɔ w'ani?	1	2	3	4	5
18.	W'ahoɔden a wode yɛ w'adwuma no nso ɛte wo sɛn?	1	2	3	4	5
19.	W'ani gye sɛdeɛ wosi teɛ ho?	1	2	3	4	5

20.	Sɛn na wosi hunu wo ne afoforɔ ntɛm nkutahodie?	1	2	3	4	5

21.	Sɛn na wani gye wɔ ɔbarima ne ɔbaa nna ho nkutahodie mu?	1	2	3	4	5
22.	Sɛn na w'ani sɔ mmoa a wonya firi wo nnamfonom hɔ no?	1	2	3	4	5
23.	W'ani sɔ w'atenaeɛ hɔ tebea hodoɔ sɛn?	1	2	3	4	5
24.	W'ani gye ɔkwan a wofa so nya ayaresa ne ayaresa ankasa a wonya ho?	1	2	3	4	5
25.	Na w'akwantusɛm nso wote nka sɛn wɔ ho?	1	2	3	4	5

Asɛmmisa a edi soɔ yi twe adwene si mpɛn dodoɔ a wate nka anaa woafa tebea hodoɔ bi mu wɔ adapɛn nnan (bosome) a atwam yi no mu?

		Ɛnsii da	Ɛwom wom a	Ɛtaa si	Mpɛn pii	Berɛ biara
26.	Mpɛn dodoɔ sɛn na wotaa nya atenka bɔne bi te sɛ woboto (w'abam bu) wobu wo ho abomfea nnoɔma haw anaa wonni anidasoɔ?	5	4	3	2	1

Wowɔ biriɔi ka wɔ saa nhwehwɛmu yi ho?

____

____

[Ɛsɛ sɛ wohyehyɛ ɛpono a edi soɔ berɛ a anototoɔ no aba n'awieeɛ no.]

		Nkotoabuo ahyɛnsodeɛ a yɛde ka mma a yɛnyaa no mmeae ahodoɔ a yɛɛyɛɛ nhwehwɛmu no	Mma yɛnyaa no tee	Mma a yesakram*	
				4-20	0-100
27.	**Nhwehwɛmu** Beaeɛ 1.	(6-Q3) + (6-Q4) + Q10 + Q15 + Q16 + Q17 + Q18<br>⑥ + ⑥ + ⑥ + ⑥ + ⑥ + ⑥ + ⑥	a. =	b:	c:
28.	**Nhwehwɛmu** Beaeɛ 2.	Q5 + Q6 + Q7 + Q11 + Q19 + (6-Q26)<br>⑥ + ⑥ + ⑥ + ⑥ + ⑥ + ⑥	a. =	b:	c:
29.	**Nhwehwɛmu** Beaeɛ 3.	Q20 + Q21 + Q22<br>⑥ + ⑥ + ⑥	a. =	b:	c:
30.	**Nhwehwɛmu** Beaeɛ 4.	Q8 + Q9 + Q12 + Q13 + Q14 + Q23 + Q24 + Q25<br>⑥ + ⑥ + ⑥ + ⑥ + ⑥ + ⑥ + ⑥ + ⑥	a. =	b:	c:

Nkekaho I: Nsɛmmisa ma Ankorɛankorɛ Amanebɔ (SRQ – 20)

DEKODE	AANE	DABI
1. Wo ti taa yɛ wo ya?		
2. W'anum ato?		
3. Wuntumi nna yie?		
4. Ɛnkyɛ na wo yam ahye wo?		
5. Wo nsa taa popo (woso) ?		
6. Wotaa bɔ hu, woyɛ senn anna nnoɔma ha wo?		
7. Wodidi a ɛtaa gyinagyina wo bo?		
8. Wo wɔ nsɛnnennen sɛ wobɛdwene ama emu ada hɔ?		
9. W'ani nnye?		
10. Wotaa susu sɛdeɛ ɛnsɛ?		
11. Ɛyɛ wo den sɛ w'ani bɛgye wo daa asetena dwumadi ho?		
12. Ɛye wo den sɛ wobɛsi gyinaeɛ?		
13. Wobrɛ wɔ dwuma a dabiara da wodie mu?		
14. Wontumi nnyɛ biribi a mfasoɔ wɔ so wɔ asetena mu?		
15. Wonni nnoɔma ho anigyeɛ?		
16. Wote nka sɛ mfasoɔ biara nni wo so?		
17. Aba wo tirim pɛn sɛ kum wo ho?		
18. Berɛ biara wote nka sɛ wabrɛ?		
19. Ɛnkyɛ na wabrɛ (wahaw)?		
20. Wo yafunum taa samforo wo?		
SRQ-20 Mma a wonnyaeɛ nnyinaa (Aane Dodoɔ)		

Hospital Anxiety and Depression Scale (HADS)
(Adwenemhaw ne Abambuo Nsania)

Hyɛ adaka a ɛwɔ mmuaeɛ a ɛne sɛdea wotee nka wɔ dapɛn a atwam no di nsɛ no no nkyɛn no nso. Nkyɛ pii wɔ wo mmuaeɛ ahodoɔ no ho : deɛ ɛbɛba w'adwenem kane no na ɛyɛ.

D	A		D	A	
		**Mete nka sɛ mayɛ senn anaa "matɔ baha" :**			**Mete nka sɛ mayɛ nyaa :**
	3	Mpɛn pii	3		Ɛrekame ayɛ sɛ berɛ biara
	2	Mpɛn dodoɔ no ara	2		Mpɛn pii
	1	Ɛtoto sisi, ɛwom wom a	1		Ɛtɔ da bi a
	0	Ɛnsii da	0		Ɛnsii da
		**M'ani da so ara gye nnoɔma a berɛni na m'anigye ho no ho :**			**Mete ayamhyehyeɛ bi nka te sɛ deɛ ehu bi :**
0		Bere biara a akyinnyeɛ enni ho		0	Ɛnsi koraa
1		Ɛnyɛ beberee saa		1	Ɛwom wom a
2		Mmerɛ kakraa bi		2	Ɛtaa si
3		Ɛnte saa koraa		3	Mpɛn pii
		**Mete ayamhyehyeɛ bi nka te sedeɛ biribi bɔne bi rebesie :**			**M'ani nnye me yɔbea ho bio :**
	3	Berɛ biara a ɛnyɛ koraa	3		Akyinnyeɛ biara nni ho
	2	Aane, nanso ɛnyɛ koraa	2		Me yɔbea no mfa me ho bio te sɛdeɛ na ɛte kane no.
	1	Kakra, nanso ɛnha me	1		Ebea menhwɛ me yɔbea no yie sɛdea anka ɛsɛ sɛ meyɛ no
	0	Ɛnsii da.	0		Mehwɛ me yɔbea no yie sɛdea meyɛ no daa no.
		**Mehunu nnoɔma a ɛyɛ sere na matumi asere :**			**Mete nka sɛ ɛsɛ sɛ meyɛ biribi nti ɛmma me ho ntɔ me :**
0		Sɛdeɛ daa metumi		3	Ɛtaa si pa ara
1		Ɛnyɛ dodoɔ biara sesei		2	Ɛdɔɔso kakra
2		Nokworɛm ɛnnɔɔso		1	Ɛnyɛ dodoɔ saa
3		Ensii da		0	Ɛnsii da
		**Nsusuiɛ a ɛha adwene taa ba me tirim :**			**Mede anigyeɛ hwɛ nnoɔma ho kwan :**
	3	Berɛ biara	0		Ɛte sɛ deɛ na meyɛ dada no ara
	2	Mpɛn pii	1		Aba fam kakra kyen sedeɛ na ɛteɛ no
	1	Ɛwom wom a, nanso ɛntaa nsi	2		Ɛsua kyɛn sedeɛ na ɛteɛ no
	0	Nnakoro nnakoro bi	3		Daabi koraa
		**M'anigye :**			**Me yam tumi hye me mpofirim :**
3		Ɛnsii da		3	Ɛtaaa si pa ara
2		Ɛntaa nsi		2	Ɛtaa si nkakrakakra
1		Ɛtɔ berɛ bi a		1	Ɛntaa nsi koraa
0		Mpɛn pii		0	Ɛnsii da
		**Metumi tena se ahotoɔ mu dwodwo me ho :**			**Metumi kenkan nwoma, tie "Radio" hwɛ "TV" so dwumadie :**
	0	Akyinnyeɛ biara nni ho	0		Ɛtaa si
	1	Ɛtaa si	1		Ɛtɔ da bi a
	2	Ɛntaa nsi	2		Ɛntaa nsi
	3	Ɛnsii da	3		Ɛwom wom a

Mesrɛ hwɛ sɛ wayi nsemmisa no nyinaa no ano.

Scoring:

Total score: Depression (D) ___________ Anxiety (A) ______________

0-7 = Normal

8-10 = Borderline abnormal (borderline case)

11 - 21 = Abnormal (case)
