# Supplementary material for: Mental health and quality of life burden in Buruli ulcer disease patients in Ghana
Source: Infect Dis Poverty. 2021 Aug 17;10:109. doi: 10.1186/s40249-021-00891-8 (PMC8367773; doi:10.1186/s40249-021-00891-8)
Supplement: Supplementary file 4 — Additional file 4: This contains comparison tables of assessment of quality of life; association of mental disorder and functional limitation between participants with active and past BU infection and comparison of mental disorders between individuals with active BU infection and caregivers. [file 40249_2021_891_MOESM4_ESM.docx]

S4: Supplementary Tables

S4.1: Comparison of mental disorders between patients with active BU infection and caregivers

| **Study participants** |  | **Depression** | | | **Anxiety** | | |
| --- | --- | --- | --- | --- | --- | --- | --- |
|  | Number | Mean (SD) | 95%CI | p value | Mean (SD) | 95%CI | *P* value |
| Active infection | 26 | 4.23 (3.95) | 2.63‒2.63 | 0.19 | 6.54 (5.38) | 4.37‒8.71 | **0.012*** |
| Caregivers | 29 | 2.79 (4.07) | 1.25‒4.34 |  | 3.21 (4.11) | 1.64‒4.77 |  |

*p value <0.05

S4.2: Comparison of association between mental disorder and functional limitation between participants with active and past BU infection

|  | **Active infection**  **n=25 (%)** | | | **Past infection**  **n=29 (%)** | | |
| --- | --- | --- | --- | --- | --- | --- |
| **Limitation** | **Mental disorder**  **absent** | **Mental disorder**  **present** | **p-value** | **Mental disorder absent** | **Mental disorder present** | ***P* value** |
| **Limitation in food preparation and Eating** | | |  |  |  |  |
| Yes | 7 (28) | 13 (52) | 0.84 |  | 6 (20) | **0.036*** |
| No | 2 (8) | 3 (12) |  | 15 (52) | 4 (14) |  |
| **Limitation in Clothing and Personal Care** | | |  |  |  |  |
| Yes | 2 (8) | 8 (32) | 0.17 | 0 | 3 (10) | **0.012*** |
| No | 7 (28) | 8 (32) |  | 19 (66) | 7 (24) |  |
| **Working Limitation** | |  |  |  |  |  |
| Yes | 5 (20) | 14 (56) | 0.07 | 3 (10) | 6 (21) | **0.014*** |
| No | 4 (16) | 2 (8) |  | 16 (55) | 4 (14) |  |
| **Limitation in Mobility** | |  |  |  |  |  |
| Yes | 7 (28) | 16 (64) | **0.049*** | 1 (4) | 3 (10) | 0.066 |
| No | 2 (8) | 0 |  | 18 (62) | 7 (24) |  |

*p value <0.05

S4.3: Assessment of transformed 0‒100 scores for quality of life domains using WHOQOL tool

| **Quality of life domain** | **BUD experience** | | | | | | **Control** | |
| --- | --- | --- | --- | --- | --- | --- | --- | --- |
|  | **Active infection** | | **Past infection** | | **Caregiver** | |  |  |
|  | Mean score ±SD | 95% CI | Mean score±SD | 95% CI | Mean score±SD | 95%CI | Mean score±SD | 95% CI |
| Physical | 54±11.1 | 49.5‒58.5 | 61.3±13.5 | 56.1‒66.5 | 56±12.7 | 52.3‒60.6 | 56±11.0 | 52.2‒59.7 |
| Psychological | 57.1±15.2 | 50.9‒63.2 | 68.4±14.6 | 62.7‒74.0 | 63.4±14.9 | 58.6‒68.3 | 64.7±11.6 | 60.8‒68.6 |
| Social | 46.7±22.4 | 37.6‒55.7 | 69.1±25.1 | 59.3‒78.8 | 59.7±24.4 | 51.7‒67.7 | 66.1±19.2 | 59.7‒72.6 |
| Environmental | 50.2±13.1 | 44.9‒55.5 | 65.3±18.2 | 58.2‒72.3 | 59.3±17.5 | 53.6‒65.1 | 58.2±12.8 | 53.9‒62.6 |
